# Proximal fibular osteotomy to treat medial compartment knee osteoarthritis: Preoperational factors for short-term prognosis

**DOI:** 10.1371/journal.pone.0197980

**Published:** 2018-05-24

**Authors:** Bo Liu, Wei Chen, Qi Zhang, Xiaoli Yan, Fei Zhang, Tianhua Dong, Guang Yang, Yingze Zhang

**Affiliations:** 1 Department of Orthopaedic Surgery, the Third Hospital of Hebei Medical University, Shijiazhuang, Hebei, P.R. China; 2 Key laboratory of biomechanics of Hebei Province, Shijiazhuang, Hebei, P.R. China; Augusta University, UNITED STATES

## Abstract

**Objective:**

The purpose of this study was to determine the association between preoperational factors and patients’ short-term outcome after proximal fibular osteotomy (PFO) and to provide a basis for detailed surgical indication and patient selection.

**Methods:**

This was a retrospective study of patients undergoing PFO between January 2015 and December 2015. Preoperational clinical data including gender, age, duration of disease, visual analogue score (VAS) and American Knee Society (KSS) score were collected. The radiological factors including hip-knee-ankle angle (HKA angle), condyle-plateau angle (CP angle), Kellgren and Lawrence grade (KL grade), joint space width of both compartments and settlement value were also considered. Patients were followed for at 12 months postoperatively. Both clinical and functional KSS scores were obtained. The outcome of interest was divided into clinical outcome and functional outcome. For each, two criteria were defined: satisfaction and significant improvement. Satisfaction is characterized by a KSS clinical or functional score over 70 points (excellent and good results); significant improvement refers to an increase in KSS scores of more than 15 points. Bivariate logistic regression for the association between preoperational factors and outcomes of interest was performed. Multivariable logistic regression analyses were used to detect the independent factors affecting the outcomes.

**Results:**

A total of 84 patients and 111 knees were followed-up. Of these, 17 knees were from males and 94 were from females. The average age was 59.45±8.82 years. The average preoperational VAS score, KSS clinical and functional score were 7.08±1.41 points, 49.14±10.95 points and 44.97±17.71 points, respectively. According to KL grading, there were 17 knees of grade 2, 47 knees of grade 3, and 47 knees of grade 4. In clinical outcomes, there were 51 knees in the satisfaction group and 77 knees in the significant improvement group. In functional outcomes, 43 knees were in the satisfaction group and 76 knees in the significant improvement group. KSS clinical score (OR = 1.134, 95%CI = 1.067–1.205, P = 0.000) was the independent factor associated with clinical satisfaction. Age (OR = 1.072, 95%CI = 1.000–1.150, P = 0.048), VAS score (OR = 1.679, 95%CI = 1.041–2.706, P = 0.033), KSS clinical (OR = 1.072, 95%CI = 1.005–1.144, P = 0.034) and functional (OR = 1.100, 95%CI = 1.044–1.159, P = 0.000) score, HKA angle (OR = 1.345, 95%CI = 1.119–1.617, P = 0.002) and settlement value (OR = 7.540, 95%CI = 1.307–43.484, P = 0.024) were the independent factors associated with functional satisfaction. KSS clinical (OR = 0.905, 95%CI = 0.850–0.963, P = 0.002) score, CP angle (OR = 0.760, 95%CI = 0.593–0.973, P = 0.030) and medial joint space width (OR = 0.001, 95%CI = 0.000–0.107, P = 0.003) were the independent factors associated with significant clinical improvement; VAS score (OR = 1.582, 95%CI = 1.042–2.402, P = 0.031), KSS functional (OR = 0.888, 95%CI = 0.838–0.942, P = 0.000) score, HKA angle (OR = 1.292, 95%CI = 1.101–1.518, P = 0.002) and settlement value (OR = 9.990, 95%CI = 1.485–67.197, P = 0.018) were the independent factors associated with significant functional improvement.

**Conclusions:**

The independent factors affecting postoperative clinical outcome after PFO were KSS clinical score, CP angle and medial joint space width. In addition, the independent factors that influenced functional outcome included age, VAS score, KSS score, HKA angle and settlement value. As objective radiological evidence, HKA angle and settlement value could be used as an important basis for patient selection for PFO.

## Introduction

Proximal fibular osteotomy (PFO) is an alternative treatment to high tibial osteotomy (HTO) [1]. It is a surgical procedure for medial compartment knee osteoarthritis (KOA). Compared to HTO, PFO has several advantages [2]. First, the surgical technique is simple and easily performed. Second, it is less invasive with a very short incision, requires limited tissue dissection and no internal fixation is implanted. The postoperative recovery period is also shorter than with HTO. In addition, the complications associated with HTO can be a major problem that contributes to a poor prognosis [3,4]. In contrast, PFO is associated with few complications [5]. Similar to HTO, PFO can relieve the symptoms of KOA with realignment of the lower extremity. However, while correcting the alignment is the major objective of HTO [6], the principle of fibular osteotomy originates from the theory of “non-uniform settlement” [7], which suggests a significant settlement of the tibial plateau following osteoporosis. As a consequence of the support by the fibula, “non-uniform” means that the settlement of the plateau is asymmetric, with settlement in the medial plateau more obvious than in the lateral plateau. Eventually, the medial plateau becomes significantly lower than the lateral and a varus deformity occurs in the lower extremity. These changes of the mechanical axis lead to a stress concentration in the medial compartment and degeneration of the cartilage and meniscus, which are the major pathological manifestations of KOA [7,8]. Thus, in “non-uniform-settlement of the tibial plateau”, KOA could also be called a “stress imbalanced syndrome of the knee joint”. PFO weakens the lateral fibular support and leads to a correction of the varus deformity, which can subsequently shift the loading force from the medial compartment more laterally, leading to decreased pain and a satisfactory functional recovery [1]. PFO is a comparatively simple surgical procedure compared to HTO. Briefly, a short lateral incision is made; between the peroneus and soleus, the muscles are then separated and the fibula is exposed. Finally, a 2-cm section of the fibula is removed 6 to 10 cm below the fibular head. Currently, the indication for PFO is medial compartment KOA [1]. However, there has been no detailed study regarding which patients would achieve a superior prognosis after PFO. In this study, to provide a basis for detailed surgical indication and patient selection, the association between preoperational factors that might affect the prognosis of PFO and patients’ outcomes is retrospectively analyzed.

## Materials and methods

### Study population

Patients with primary medial compartment KOA who had an indication for PFO admitted to our hospital from January to December, 2015, were retrospectively analyzed in this study. The patients were all diagnosed according to the criteria of the American College of Rheumatology [9]. Inclusion criteria were patients with moderate to severe symptoms of the knee over Kellgren and Lawrence (KL grade) grade 2 on radiographs [10]. The exclusion criteria were as follows: rheumatoid arthritis, posttraumatic arthritis, congenital deformities of the lower extremity, joint infection, history of ligament or meniscus injury and significant abnormality of the lateral compartment. The study was approved by the Ethical Committee of the Third Hospital of Hebei Medical University and was conducted in accordance with the Declaration of Helsinki.

### Clinical evaluation

Clinical data were divided into gender, age, duration of disease and grading systems. The latter included visual analogue scale score (VAS score) and American Knee Society score (KSS score), which consisted of both clinical score and function score [11]. The clinical score of KSS major includes pain, stability and range of motion. In addition, the function score focuses on the activities of the patient. Each extremity undergoing a surgical procedure was evaluated independently. Patients who underwent bilateral PFO were regarded as two independent participations. All of the information was acquired from the follow-up center of our hospital.

### Radiographic evaluation

In this study, radiological factors included hip-knee-ankle angle (HKA angle) [12], condyle-plateau angle (CP angle) [13], KL grade [10], joint space width of both compartments and settlement value [14,7]. The HKA angle was measured on spliced images of total length lower extremities and all other measurements were made on weight-bearing AP radiographs. The detailed methods for radiological measurements were previously described [7,10,12,13,14]. A computerized goniometer or length meter of the Synapase System (Fujifilm Medical Systems, Stamford, U.S.A.) was used for measurement. To test the intra-and inter-observer reproducibility, two orthopedic surgeons performed all of the radiographic measurements in 30 randomly selected patients. The measurements were repeated after one week. The intra-class correlation coefficient (ICC) was used for assessment of the intra-and inter-observer reliabilities. The results showed good reliability (ICC>0.9 in each measurement).

### Surgical technique

The surgical technique has been previously described [1]. Briefly, a lateral incision of 3 to 5 cm was made at the proximal third of the fibula. The fascia was then incised in line with the septum between the peroneus and soleus, the muscles were separated, and the fibula was exposed. A 2-cm section of the fibula was removed 6 to 10 cm below the fibular head with the use of an oscillating saw or fret saw. Following resection, the fibula ends were sealed with bone wax. The muscles, fascia, and skin were then sutured separately. For each patient of the study, the operation was done by the same surgeon (Y Zhang). Postoperatively, the patients ambulated as soon as the pain could be tolerated.

### Follow-up

Patients were followed up at 12 months postoperatively. The clinical and functional KSS scores were obtained.

### Outcome

The outcome of interest was divided into clinical outcome and functional outcome. For each, two criteria were defined: satisfaction and significant improvement. Satisfaction is characterized by a KSS clinical or functional score over 70 points (excellent and good results); significant improvement refers to an increase in KSS scores of more than 15 points.

### Statistical analyses

Statistical analyses were performed using SPSS version 19.0 statistical software for Windows (IBM, Armonk, New York). Continuous variables were expressed as the mean±SD and categorical variables were expressed as frequencies. Bivariate logistic regression analyses were used to assess the association between preoperational factors and outcomes of interest. Multivariable logistic regression analyses were used to detect the independent factors affecting the outcomes. A P value less than 0.05 was considered to be significant.

## Results

A total of 88 patients were included in this study. Two patients quit the study for their personal reasons and two patients were lost to follow-up. There were 84 patients and 111 knees available. All patients were evaluated one year (two weeks before or after one year) postoperatively. There were 17 knees of males and 94 knees of females. The average age and duration of disease was 59.45±8.82 years (range from 43 to 86 years) and 6.57±5.37 years (range from 0.1 to 25 years), respectively. The average preoperational VAS score, KSS clinical and functional score was 7.08±1.41 points (range from 3 to 9 points), 49.14±10.95 points (range from 25 to 89 points) and 44.97±17.71 points (range from 0 to 90 points), respectively. According to KL grading, there were 17 knees of grade 2, 47 knees of grade 3, and 47 knees of grade 4. The average preoperational HKA angle and CP angle was 170.54±4.93 degrees (range from 148.77 to 179.45 degrees) and 3.78±2.99 degrees (range from 0.01 to 21.02 degrees), respectively. The average preoperational settlement value was 0.68±0.42 cm (range from -0.13 to 2.05 cm). The average preoperational medial and lateral joint space width was 0.30±0.17 cm (range from 0.04 to 0.70 cm) and 0.63±0.16 cm (range from 0.23 to 1.17 cm), respectively. The average postoperative KSS clinical and functional scores were 67.77±11.08 points (range from 32 to 95 points) and 64.66±13.12 points (range from 20 to 100 points), respectively. There were 51 knees with satisfactory clinical outcome and 77 knees with significant improvement; 43 knees had satisfactory functional outcome and 76 knees with significant improvement.

Bivariate logistic regression for the association between preoperational factors and outcomes of interest was performed (Table 1). VAS score and KSS clinical score were statistically associated with clinical satisfaction and KSS clinical and functional score; HKA angle and CP angle were statistically associated with functional satisfaction. Age, VAS score, KSS clinical score, KL grade and medial joint space width were statistically associated with significant clinical improvement and KSS functional score was statistically associated with significant functional improvement.

**Table 1:** Odds ratios of Bivariate logistic regression analyses for outcome of interest versus patient clinical and radiological characteristic.

Characteristic	Satisfaction in clinical outcome			Satisfaction in functional outcome			Significant improvement in clinical outcome			Significant improvement in functional outcome		
	OR	95%CI	P	OR	95%CI	P	OR	95%CI	P	OR	95%CI	P
Gender: Male vs. Female(ref)	0.718	0.255–2.022	0.531	0.667	0.236–1.888	0.446	2.325	0.810–6.671	0.117	1.650	0.570–4.773	0.355
Age	0.992	0.950–1.037	0.737	0.993	0.949–1.039	0.759	1.079	1.021–1.140	0.007	1.039	0.988–1.092	0.134
Duration of disease	0.957	0.889–1.029	0.234	0.966	0.896–1.041	0.362	1.089	0.994–1.191	0.066	1.056	0.972–1.148	0.198
VAS Score	0.656	0.485–0.887	0.006	1.071	0.813–1.412	0.626	1.460	1.085–1.963	0.012	1.305	0.981–1.736	0.067
KSS Clinical Score	1.110	1.055–1.168	0.000	1.064	1.020–1.109	0.004	0.897	0.848–0.949	0.000	0.969	0.932–1.006	0.102
KSS Functional Score	1.003	0.982–1.025	0.768	1.093	1.046–1.142	0.000	0.980	0.955–1.005	0.116	0.904	0.860–0.950	0.000
K-L Grade	1.016	0.600–1.719	0.954	0.606	0.351–1.046	0.072	1.827	1.027–3.250	0.040	0.956	0.543–1.684	0.876
HKA Angle	1.048	0.969–1.135	0.242	1.153	1.046–1.270	0.004	0.959	0.879–1.046	,0.343	1.020	0.941–1.106	0.627
CP Angle	0.985	0.868–1.117	0.810	0.831	0.705–0.978	0.026	1.040	0.901–1.200	0.593	0.996	0.871–1.139	0.954
Settlement Value	0.594	0.235–1.503	0.271	0.534	0.202–1.412	0.206	1.721	0.615–4.816	0.301	1.919	0.682–5.397	0.217
Medial Joint Space Width	0.623	0.070–5.512	0.670	6.540	0.680–62.911	0.104	0.018	0.001–0.236	0.002	0.471	0.046–4.830	0.526
Lateral Joint Space Width	0.217	0.020–2.406	0.213	0.075	0.005–1.061	0.055	0.624	0.052–7.428	0.709	0.618	0.053–7.229	0.701

OR, odds ratio; CI, confidence interval for odds ratio; VAS, visual analogue score; KSS, American Knee Society score; KL grade, Kellgren and Lawrence grade; HKA angle, hip-knee-ankle angle; CP angle, condyle- plateau angle

Multivariate logistic regression analysis revealed that the odds for clinical satisfaction approximately increased by 13.4% for every point of KSS clinical score increase (OR, 1.134; 95%CI, 1.067–1.205). The odds for functional satisfaction approximately increased by 7.2% for every year of age increase (OR, 1.072; 95%CI, 1.000–1.150), by 67.9% for every point of VAS score increase (OR, 1.679; 95%CI, 1.041–2.706), by 7.2% for every point of KSS clinical score increase (OR, 1.072; 95%CI, 1.005–1.144), by 10% for every point of KSS functional score increase (OR, 1.100; 95%CI, 1.044–1.159), by 34.5% for every degree of HKA angle increase (OR, 1.345; 95%CI, 1.119–1.617) and by 654% for every centimeter of settlement value increase (OR, 7.540; 95%CI, 1.307–43.484). The odds for significant improvement in clinical outcome approximately decreased by 9.5% for every point of KSS clinical score increase (OR, 0.905; 95%CI, 0.850–0.963), by 24% for every degree of CP angle increase (OR, 0.760; 95%CI, 0.593–0.973) and by 99.9% for every centimeter of medial joint space width increase (OR, 0.001; 95%CI, 0.000–0.107). The odds for significant improvement in functional outcome approximately decreased by 11.2% for every point of KSS functional score increase (OR, 0.888; 95%CI, 0.838–0.942), but increased by 58.2% for every point of VAS score increase (OR, 1.582; 95%CI, 1.042–2.402), by 29.2% for every degree of HKA angle increase (OR, 1.292; 95%CI, 1.101–1.518) and by 899% for every centimeter of settlement value increase (OR, 9.990; 95%CI, 1.485–67.197) (Table 2).

**Table 2:** Odds ratios of Multivariate logistic regression analyses for outcome of interest versus patient clinical and radiological characteristic (only variables in equation are shown in the table).

Characteristic	Satisfaction in clinical outcome			Satisfaction in functional outcome			Significant improvement in clinical outcome			Significant improvement in functional outcome		
	OR	95%CI	P	OR	95%CI	P	OR	95%CI	P	OR	95%CI	P
Gender: Male vs. Female(ref)							4.105	0.089–18.954	0.07			
Age				1.072	1.000–1.150	0.048	1.068	0.995–1.146	0.068			
VAS Score				1.679	1.041–2.706	0.033				1.582	1.042–2.402	0.031
KSS Clinical Score	1.134	1.067–1.205	0	1.072	1.005–1.144	0.034	0.905	0.850–0.963	0.002			
KSS Functional Score	0.975	0.948–1.004	0.086	1.1	1.044–1.159	0				0.888	0.838–0.942	0
HKA Angle				1.345	1.119–1.617	0.002				1.292	1.101–1.518	0.002
CP Angle							0.76	0.593–0.973	0.03			
Settlement Value				7.54	1.307–43.484	0.024				9.99	1.485–67.197	0.018
Medial Joint Space Width							0.001	0.000–0.107	0.003			

OR, odds ratio; CI, confidence interval for odds ratio; VAS, visual analogue score; KSS, American Knee Society score; KL grade, Kellgren and Lawrence grade; HKA angle, hip-knee-ankle angle; CP angle, condyle- plateau angle

## Discussion

Previous studies have shown that PFO could be used as an effective treatment for KOA, which can significantly improve the hip-knee-ankle angle and KSS score of postoperative patients [1]. However, the short-term (one-year) postoperative clinical situation improvements of some patients remained unsatisfactory [5]. Therefore, how to improve the outcome of PFO is an important issue. In contrast to HTO, which has a variety of surgical methods and whose angle of osteotomy could be controlled, the number of PFO surgical methods is less, so patient selection has become a crucial factor for further identification of the operative indications. The objective of this study was to observe the effects of preoperative clinical and radiographic factors on short-term postoperative outcome to guide the selection of surgical patients, to further clarify the indications for surgery and to improve postoperative curative effect. In this study, VAS and KSS scores were selected as clinical evaluation factors. In addition, radiographic factors were selected, including KL grade, HKA angle, CP angle, and joint space width. Settlement value [7], a factor reflecting the degree of non-uniform-settlement of the tibial plateau, was also included. This study mainly focused on factors influencing the outcome of the operation, so it was rather important to select the appropriate follow-up time. To date, there has been no report on the outcome and postoperative time of PFO. Taking HTO as a reference, some reports stated that the clinical situation of patients one year after the operation trended towards stability [15]. Therefore, it was chosen as the follow-up time. It is believed that follow-up at this time could demonstrate whether the operation succeeded or failed. Symptoms of KOA might reappear and become progressively aggravated as time progressed [16], which was not taken into consideration in this study and will be considered in the future study.

The study results indicated that preoperative KSS clinical score was the sole independent factor associated with the clinical satisfaction of patients. This does make sense, as it was apparently easier for patients suffering less severe disease to achieve satisfactory results. Meanwhile, there are a number of independent factors that can significantly improve patients’ clinical outcome, such as KSS clinical score, CP angle and medial joint space. Apart from the ones with lower preoperative clinical scores, patients with obvious medial space narrowing and smaller CP angle were more likely to achieve significant improvements in clinical symptoms. The next two factors were usually associated with articular cartilage [17,18]. Due to the stress concentration in the medial compartment, cartilage was worn and degenerated under sustained pressure [19], leading to medial space narrowing in patients with KOA. The stress concentration might be associated with the non-uniform-settlement of the tibial plateau and the support of the fibula [1]. Therefore, after removal of the cause (referring to the PFO), patients’ clinical symptoms could be improved to a large extent. Moreover, advanced medial space narrowing was related to the severity of the disease [20]. For patients with KOA of great severity, it is difficult to achieve an excellent-to-good result of clinical outcome, but there is more room for a significant improvement (KSS change >15).

In terms of functional evaluation, age, VAS score, KSS clinical and functional scores, HKA angle and settlement values were all independent factors affecting satisfactory functional outcome. For significant improvement of outcome, the results were similar. HKA angle reflected the changes in limb alignment [21], and patients with nearly normal HKA angles showed better outcomes in joint function, which might be because PFO could only partially correct the varus deformity of the tibial plateau. Studies have shown that patients with severe KOA had varus deformity in the femoral condyle as well [22]. For these patients, PFO was unable to fully improve their varus deformity and prognosis. In addition, settlement value was taken as a factor to reflect the degree of non-uniform-settlement of the tibial plateau [7]. The higher the settlement value, the more significant the effect of lateral fibula support and the better the outcome of PFO. Such findings suggested that PFO in the treatment of KOA was closely related to the non-uniform-settlement theory. Patients with higher settlement value undergoing PFO operation could be expected to obtain better functional outcome.

Of the factors associated with the outcome of PFO, medial joint space, CP angle, HKA angle and settlement value were objective factors and could be measured directly on X-ray films. Therefore, these factors were not subject to subjective impact, and thus suitable for prediction of a patient's postoperative recovery.

The limitations of this study were as follows. First, the sample size was relatively small. Second, the follow-up time was short, making us unable determine the relationship between study factors and long-term postoperative outcome of PFO. Furthermore, this study only took preoperative factors into account, and failed to consider the impact of the operation itself and postoperative recovery on outcomes.

## Conclusions

In conclusion, KSS clinical score, CP angle and medial joint space width were the independent factors affecting postoperative clinical outcome after PFO. In addition, independent factors influencing functional outcome included age, VAS score, KSS score, HKA angle as well as settlement value. In particular, as objective evidence of radiography, HKA angle and settlement value were less affected by subjective factors and were easy to measure. Therefore, these two factors could be used as the main bases for patient selection.
